# Modern Medico-Psychology and Psychiatry

**Published:** 1894-10-06

**Authors:** T. S. Clouston

**Affiliations:** Lecturer on Mental Diseases, Edinburgh University, and Physician Superintendent Royal Asylum, Morningside


### Modern Medico-Psychology and Psychiatry.

By T. S. Clouston, M.D., Lecturer on Mental Diseases, Edinburgh University, and Physician Superintendent Royal Asylum, Morningside.

### XII.—MENTAL STATES OF ALTERNATION, RHYTHM, AND PERIODICITY.

I have indicated the chief symptoms of the two great conditions of mental depression and mental exaltation—melancholia and mania. Taking recent insanity and first attacks, these cover 80 per cent. of all the cases; and I think in frequency they are about equal, if the whole field of mental disease is looked at. Of hospital insanity, no doubt mania covers two-thirds and melancholia only one-third; but a different proportion is seen if the whole prevalence of the disease is taken into account. The slighter cases of depression are more manageable, more reasonable, more able to go on doing work, and are not, therefore, sent to institutions. They are not tabulated, except in the private notes of the family doctor, when he keeps them. In consulting mental practice melancholia is the most frequent of all the ailments the doctor sees. Where cases are observed through their whole course, the physician finds that very many of them change in character, the depressed becoming elevated, and the elevated becoming depressed. Further study shows that many of them develop a more or less regular tendency towards an alternation of different mental conditions, towards a rhythm of mental changes, and a periodicity of recurrence of the same conditions over and over again. A careful study of the cases of mental disease I have had under treatment shows that at least one-half of all the recent cases of depression and elevation had relapses, or recurrences, or alternations of mental symptoms during the attacks. Such a study shows, too, that these changes have different characters in different cases. In one we see a diurnal alternation, the patient being elevated one day and depressed or well the next, this going on for some time. Another will have a morning depression and an evening remission. This is, indeed, very common in melancholia, especially in its early and later stages, when it is coming on and going off. Others have remissions and alternations lasting a few days. In others, again, the relapses are marked off from each other by periods of four weeks, and in a few the cycle is seasonal. No doubt it was the four-weekly cases that originated the idea that insanity had a relationship to the phases of the moon, and gave the name "lunacy" to the disease. There is not a shadow of proof that the moon affects in any way the mental condition of any man or woman in health or disease. But our nature-worshipping and observing ancestors, seeing the great lunar phenomena of the tides, and seeing also that many mental cases had a four-weekly aggravation, correlated the two phenomena. Such false beliefs die hard.

There can be no doubt that the periodicity of mental symptoms in disease is a pathological expression and aggravation of the physiological periodicities of the organism, of the universal mental and bodily brain rhythm of sleep and waking, of the reproductive periodicities of all the higher animals, and of the seasonal periodicities of some of them. Normal mind is periodic in action, therefore abnormal mind is still more so; and the accentuation of normal mental action in specially unstable brains becomes mania, while the aggravation of normal reaction becomes melancholia. Many brains show their hereditary instability by the morbid accentuation of the normal. This is another illustration of the law that all disease is only altered health, that pathological changes in structure and function are only accentuated physiological changes. Insanity is not a new something that enters into a man. It is only an aggravation of what is in him already. Let any man calmly consider his mental state in the middle of the night, and compare it with his state in broad daylight, and he will see that he has not far to go to seek one origin of some of the delusions, the diminished volition and courage, and the morbid fears of the melancholic. The fact is that almost every man and woman has two mental lives to some extent, the one assumed by day and the other by night. Did not Macbeth, who "was never taint with fear" by day, succumb at night to abject terror and hullucinations?

> Mine eyes are made the fools o' th' other senses.
> How is't with me when every noise appals me?

It may be said that conscience made a coward of Macbeth, as it does of many men; but night as well as conscience makes cowards of us all. This, with sleep, is the great illustration of the diurnal periodicity of mind.

There is a special form of mental disease first described in France, whose definite character is given to it by its periodicity, and hence it is called "Folie Circulaire." In it there are three sections of the mental circle that the patient moves in—viz., depression, elevation, and sanity; and in this round he spends his life, passing out of one into the other, for it is, when fully established, a very incurable disease. The patient takes an attack of mania, during which he is joyous, restless, troublesome, extravagant, and often vicious. He eats voraciously, sleeps little, and never seems to tire. His temperature is a degree or so above the normal; his eye is bright and glistening; he is enamoured of the other sex; he shows diminished self-control, and no common sense. This lasts for a few weeks, or a few months more commonly, and then he passes sometimes gradually and sometimes rather suddenly into a condition of depression during which he is sluggish, dull, looking differently, dressing differently, eating differently, fearful, unreliant, and sedentary in habits. This state will last a few weeks or months, and the patient will brighten up into what seems recovery, and is to all intents and purposes in his normal state. This, again, lasts for a few weeks or months, and he gradually gets morbidly elevated. You find he is passing through every minute mental phase and habit he did at first; depression follows as before, and then sanity; and this round of three states of feeling, of intellect, of volition, and of nutrition, goes on, circle after circle, till the patient dies. He lives three lives. When such patients are studied, it is found in typical cases that the circles tend to enlarge as time goes on. I had one such case, whose circle of the three stages took six months to complete when he was forty-six, at sixty-two took six years. This singular and most interesting disease is best and most typically seen in members of old and distinguished families who have become tainted with insanity. A few such cases recover. A few pass into stupor and weak-mindedness, but the majority go on in their alternations till death.

A wise and experienced doctor always prepares the minds of relatives in certain cases for relapses in insanity. Certain drugs are found very useful in arresting these periodic and rhythmical morbid tendencies. The most notable of these are the bromides and sulphonal. The latter drug I found to arrest the recurrent tendency that had lasted in one case for over twenty years.

